# Emerging Role of Spinal Cord TRPV1 in Pain Exacerbation

**DOI:** 10.1155/2016/5954890

**Published:** 2016-01-14

**Authors:** Seung-In Choi, Ji Yeon Lim, Sungjae Yoo, Hyun Kim, Sun Wook Hwang

**Affiliations:** ^1^Department of Biomedical Sciences, Korea University College of Medicine, Seoul 136-705, Republic of Korea; ^2^Department of Physiology, Korea University College of Medicine, Seoul 136-705, Republic of Korea; ^3^Department of Anatomy, Korea University College of Medicine, Seoul 136-705, Republic of Korea

## Abstract

TRPV1 is well known as a sensor ion channel that transduces a potentially harmful environment into electrical depolarization of the peripheral terminal of the nociceptive primary afferents. Although TRPV1 is also expressed in central regions of the nervous system, its roles in the area remain unclear. A series of recent reports on the spinal cord synapses have provided evidence that TRPV1 plays an important role in synaptic transmission in the pain pathway. Particularly, in pathologic pain states, TRPV1 in the central terminal of sensory neurons and interneurons is suggested to commonly contribute to pain exacerbation. These observations may lead to insights regarding novel synaptic mechanisms revealing veiled roles of spinal cord TRPV1 and may offer another opportunity to modulate pathological pain by controlling TRPV1. In this review, we introduce historical perspectives of this view and details of the recent promising results. We also focus on extended issues and unsolved problems to fully understand the role of TRPV1 in pathological pain. Together with recent findings, further efforts for fine analysis of TRPV1's plastic roles in pain synapses at different levels in the central nervous system will promote a better understanding of pathologic pain mechanisms and assist in developing novel analgesic strategies.

## 1. Introduction

Transient receptor potential vanilloid subtype 1 (TRPV1) is a well-known pain-mediating ion channel expressed in sensory neurons including dorsal root ganglionic (DRG) neurons, trigeminal ganglionic (TG) neurons, and vagal neurons. In response to various harmful stimuli, TRPV1 pore opens and cationic flux through the pore into the nerve terminal causes electrical depolarization which may lead to action potential generation. When propagated and transmitted into the brain, the signals finally result in the perception of pain. Because of its extreme polymodality compared to other known peripheral sensor molecules that allows TRPV1 to cover a large spectrum of pain qualities from chemical through thermal ones and of its famous activator capsaicin which has been traditionally utilized for pain research even before TRPV1 discovery, TRPV1 has garnered a great deal of attention as a peripheral pain-modulating target. Topical application of a TRPV1 modulator is currently the mainstream for TRPV1-targeting analgesic strategies while systemic approaches have been dropped due to a potential for hyperthermic adverse effect because body temperature regulation is perturbed by antagonism of vagal TRPV1.

Although the initial report about TRPV1 finding suggested that it is specifically expressed in sensory neurons, its wider distribution in various regions including certain regions of the central nervous system (CNS) and nonnervous tissues has been surmised (for review, [1]). Using rodent and human brains, Mezey et al. verified the existence of TRPV1 protein and mRNA in the spinal cord, amygdala, medial and lateral habenula, hippocampus, striatum, hypothalamus, centromedian and paraventricular thalamic nuclei, substantia nigra, reticular formation, locus coeruleus, cerebellum, inferior olive, and certain cortical areas [2]. Soon after, Valtschanoff et al., focusing on the spinal cord, showed that both presynaptic (from the central terminal of sensory neurons) and postsynaptic regions (from the dendrites of spinal cord dorsal horn neurons) exhibit TRPV1 positivity, especially in the superficial laminae I and II, which are the first relaying stations in the pain sensory pathway [3]. Using dorsal rhizotomy, they were able to histologically show that postsynaptic TRPV1 expression levels were highly dependent on the presence of peripheral inputs, indicating that spinal cord TRPV1 expression and function may be dynamically controlled by sensory states. Since that time, CNS and spinal cord expression of TRPV1 have been persistently confirmed [4, 5]. These results suggested that TRPV1 may play a role in the central areas and in this review we more focus on pain transmission in the spinal cord.

## 2. TRPV1 Expression in Neuropathic Pain Models

Altered expression of a protein depending on disease states often implies its importance in disease progression. Upregulation of TRPV1 in DRG or TG neurons under a proinflammatory state has been reported [6–9]. Differential regulation of TRPV1 expression occurs in neuropathic pain states. At the scale of whole DRG neuronal collection, including both damaged and undamaged neurons, the amount of TRPV1 was reduced in many different neuropathy models including those of sciatic nerve axotomy [10], partial nerve ligation [11], chronic constriction injury (CCI) [12], spinal nerve ligation [13], and diabetic neuropathy [14, 15]. The loss of total TRPV1 expression appears to be at least partially due to the degeneration of damaged TRPV1-positive DRG neurons. Interestingly, in the spinal cord dorsal horn, TRPV1 is upregulated in a CCI neuropathic pain model [16]. When looking at uninjured DRG neurons, higher TRPV1 expression was detected even in the neurons at different spinal levels from that for damaged ones [11, 17]. Different from the peripheral neuropathy models mentioned above, in the spinal cord injury models, an increase in TRPV1 proteins or mRNAs was consistently detected in the DRG [18–20]. The molecular and cellular mechanisms for such increased TRPV1 expressions in undamaged neurons in diverse injury models remain undetermined and we dealt with those in “Unsolved Issues” below. Briefly, the uninjured DRG neurons may be affected by inflammatory processes, for example, via increased secretion of inflammatory mediators such as nerve growth factor (NGF) from recruited immune components around adjacent damaged DRG or spinal cord regions. Indirect synaptic mechanisms via collaterals or descending circuits also likely participate in TRPV1 upregulation in DRGs at different spinal levels. The results of elevated TRPV1 levels indicate that peripheral TRPV1 expression can be controlled upon injury conditions and that increased amplification of pain signals may involve upregulation of TRPV1, which might serve as a potential leverage for therapeutic modulation.

## 3. Insights from Spinal TRPV1 Antagonism

Besides expression results, outcomes from pharmacological manipulation of spinal cord TRPV1 activity have also consistently emphasized its crucial role in pain transmission and therapeutic advantages. In particular, industrial field hypothesized that selective antagonism to spinal TRPV1 could be one option for avoiding adverse malignant hyperthermia since CNS TRPV1 seems to be free from the hyperthermic mechanism [21, 22]. Kanai et al. of Pfizer Japan not only demonstrated increased TRPV1 levels in the spinal cord of CCI rats but also produced a promising analgesic result from intrathecal administrations of the TRPV1 antagonist BCTC [16]. In the hundreds of nanomolar range, mechanical allodynia and calcitonin gene-related peptide-like immunoreactivity and substance P-like immunoreactivity were attenuated in the spinal cord from the CCI-injured rats [16]. Researchers at Abbott Labs used three different inflammatory pain models with complete Freund's adjuvant, capsaicin, and sodium monoiodoacetate injections. Their CNS-penetrable version, A-784168, more effectively blocked pain than a less penetrable A-795614, despite being similar in terms of* in vitro* profiles for TRPV1 antagonism [23]. Watabiki et al. at Astellas Pharma demonstrated that mechanical allodynia in their mouse spinal nerve ligation (SNL) model was alleviated by intrathecal injection of either of BCTC or their own TRPV1 antagonist AS1928370 [24]. A little later, the paradigm was also confirmed in the academic field. Wu et al. demonstrated that TRPV1 antagonism using intrathecal AMG9810 reversed mechanical and thermal hypersensitivities in a contusive spinal cord injury model [20]. Spinal TRPV1 knockdown with antisense oligonucleotide produced similar results. In fact, approaches with agonists have provided a similar insight. Two research groups independently demonstrated that intrathecal agonist (capsaicin or 9-hydroxyoctadecadienoic acid) injections induced mechanical allodynia [25, 26]. Since TRPV1 is not a mechanosensitive ion channel and thus TRPV1 in the periphery has no role in mediating mechanical phenotypes, the mechanical hypersensitivity is purely due to central TRPV1 activity on transmission. An agonist was used for the opposite purpose [27, 28]. Intrathecal injections of resiniferatoxin (RTX), a potent TRPV1 agonist, deactivated voltage-dependent components or ablated TRPV1-positive neuronal terminals [29–31]. Interestingly, in a carrageenan-induced inflammation model, the thermal threshold but not the mechanical threshold was normalized with this strategy [27, 28]. Although the model involved an agonistic challenge, body temperatures of the treated animals were largely unaffected. Collectively, the results from expression dynamics and pain pharmacology commonly raise the importance of the existence of spinal cord TRPV1. In turn, several groups have begun to explore the next question: how, differently from peripheral TRPV1, spinal TRPV1 intervenes pain transmission in pathologic states.

## 4. Proposed Nociceptive Roles of TRPV1 in the Spinal Cord

Spinal synaptic plasticity is a central concept that accounts for pathologic transition from a normal acute pain to a chronically morbid one [32]. Both long-term potentiation and depression (LTP and LTD) paradigms and technical progress for brain slice electrophysiology from learning and memory research mostly using the hippocampal area were imported to the pain field. Those concept and technology have contributed to the understanding of the pathological pain transmission in the spinal cord and to finding painkilling targets: ionotropic and metabotropic glutamate receptors, N-type and T-type voltage-gated Ca^2+^ channels, upstream and downstream signaling molecules of the nitric oxide synthesis pathway, calcium/calmodulin-dependent kinases, neuropeptides and their receptors, and so forth [33]. For validating the spinal TRPV1 mechanism, this advantage has begun to be utilized. In this context, three important aspects of the roles of spinal TRPV1 have been reported in recent years [26, 34, 35]. The discussions about the detailed results follow.

### 4.1. Presynaptic TRPV1: Excitatory

Gone through simple traditional observations where spontaneous excitatory postsynaptic currents (sEPSCs) were facilitated by the presynaptic actions of capsaicin to understand the circuitry under normal conditions [36, 37], researchers became interested in TRPV1's role in pathologic states. Xu et al. found that TRPV1 in the central terminal of DRG neurons plays an important role in exaggerating pain in an inflammatory state during their analyses of the effects of endogenous proresolving lipids [34]. When they recorded the lamina II neurons of transverse slices of the murine lumbar spinal cord with a patch clamp technique, sEPSCs were increased upon tumor necrosis factor-*α* (TNF-*α*) exposure. Because the frequency but not the amplitude of EPSCs was increased, TNF-*α* seemed to elevate glutamate release by acting at presynaptic terminals. This perfusion with TNF-*α ex vivo* may represent an inflammatory or neuropathic pain state* in vivo*. Simultaneous treatment of capsazepine reversed this TNF-*α* effect: the changes were limited to numbers of sEPSC frequencies. Consequently, the results of this study provide multiple implications: the primary action site of the acute TNF-*α* effect is presynaptic sensory neurons; TRPV1 had been presumed to be a major effector of TNF-*α* action, at least in the periphery [38, 39], and the results confirmed it and extended to the central terminals; regarding capsazepine-affected parameters, TRPV1-mediated mechanism may be more important in presynapses. Despite being out of the scope of this review, resolvin E1, a potent endogenous proresolving lipid, appears to disturb the presynaptic signaling between TNF-*α* and TRPV1 via its G-protein-coupled receptor (GPCR) activation, as a part of its painkilling mechanisms [40–42].

### 4.2. Presynaptic TRPV1: Receiving Descending Excitatory Input

More recently, the Wei and Dong labs revisited the presynaptic role of TRPV1 from the viewpoint of descending excitatory modulation when they tried to explain secondary hyperalgesia that occurs from neighboring but uninjured receptive field under neuropathic conditions [35]. They developed a knock-in mouse line in which the expression of GCaMP3 encoding a Ca^2+^ indicator protein is driven by the promoter of Pirt (phosphoinositide-interacting regulator of transient receptor potential channels), a pan-DRG/TG marker [43]. With this mouse model, changes in intracellular Ca^2+^ levels in cell bodies and peripheral and central terminals can be discerned in DRG and TG neurons, even under* ex vivo* conditions surrounded by other tissue types or buried in complex synaptic circuits. They also created a cheek mechanical hyperalgesia model using CCI of the infraorbital nerve, which is the major branch of the maxillary (V2) TG nerve. Accordingly, the trigeminal subnucleus caudalis (Vc), which is analogous to the spinal dorsal horn in terms of the sensory synaptic circuit, was observed for presynaptic TRPV1 functions.

**Figure 1:**
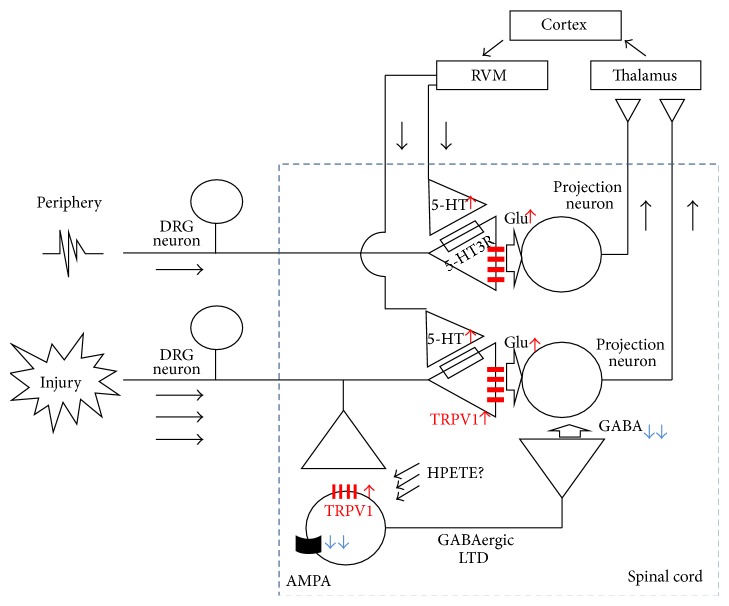
Schematic representation of the role of TRPV1 for pain exacerbation in the spinal cord pre- and postsynapses.

Although only V2 TG nerve had undergone the CCI procedure, heightened pain sensitivities occurred in the cheek, jaw, and ear, the latter two of which are mandibular (V3) TG nerve territories. When intracellular Ca^2+^ fluorescence level due to GCaMP3 was analyzed as a surrogate measure for excitability, both V2 and V3 central terminals in Vc exhibited larger Ca^2+^ increases than under normal conditions. Furthermore, these elevated sensitivities were commonly observed in terminals from superficial through deep laminae, suggesting that not only injured but also adjacent undamaged nerve fibers became hyperactive and that this situation consequently led to secondary hyperalgesia and allodynia. Based on their previous observations, Wei and Dong's group hypothesized that the rostral ventromedial medulla (RVM) in the brainstem relays 5-hydroxytryptamine (5-HT, serotonin) dependent excitatory input to uninjured nerves [44, 45]. Indeed, 5-HT immunoreactivity was elevated near the GCaMP3-positive central sensory terminals of Vc. Moreover, antagonistic manipulations including treatment using the 5-HT3 receptor antagonist in Vc or 5-HT depletion in RVM using RNA interference against its biosynthesis alleviated both hyperactivity of TRPV1-mediated Ca^2+^ signals and hypersensitive behaviors. Since 5-HT receptor-mediated TRPV1 facilitation was confirmed in the central presynaptic terminals, the descending excitatory axons from RVM seem to constitute axoaxonal contacts. Collectively, TRPV1 in the presynapse of sensory neurons that covers the undamaged regions participates in secondary pain amplification through a descending facilitation mechanism (Figure 1).

### 4.3. Postsynaptic TRPV1: Disinhibiting Secondary Projection

Previous positive results of the spinal cord expressions of TRPV1 strongly implicated a functional role of TRPV1 in the spinal postsynaptic neurons [2–4]. The Oh lab focused on the spinal cord inhibitory synapse regarding the role of TRPV1. In fact, loss of GABAergic or glycinergic inhibitory control in the spinal synaptic network has long been proposed as a cause of central pain sensitization [46–48]. Different from sEPSCs, evoked EPSCs have been reported to decrease after capsaicin perfusion [49, 50]. TRPV1 activation acutely induced increases in frequency in spontaneous inhibitory postsynaptic currents (sIPSCs) in the dorsal horn neurons via GABAergic or glycinergic connections [51, 52]. Oh's group hypothesized that TRPV1 plays a role in the pathologic condition. As mentioned, intrathecal injection of capsaicin elicits mechanical allodynia [26]. Notably, in a mouse model with ablation of TRPV1-positive DRG neurons, a significant proportion of mechanical hypersensitivity remained following intrathecal capsaicin administration, indicating that postsynaptic TRPV1 also contributes to the pain state. Indeed, TRPV1 expression and agonist-dependent activation in postsynaptic dorsal horn neurons were verified, and over 75% of GABAergic interneurons were TRPV1-positive whereas ~75% of non-GABAergic postsynaptic neurons were TRPV1-negative. Surprisingly, in their EPSC profiling of the GABAergic neurons in response to electrical stimulation of the dorsal root entry zone, LTD occurred after postsynapse-specific TRPV1 activation. TRPV1 activation-induced LTD was dependent on an increase in the intracellular Ca^2+^ concentration and the reduction of alpha-amino-3-hydroxy-5-methyl-4-isoxazolepropionic acid (AMPA) receptor activities, similar to other typical LTD processes. Accordingly, LTD caused less excitability and reduced GABA release of the GABAergic interneurons, and therefore the secondary projection neurons (spinothalamic tract neurons in this study) received less inhibitory input, resulting in enhanced relay of pain signals to higher brain areas (Figure 1). The analgesic effects of spinal TRPV1 antagonism in mice CCI model were successfully repeated without malignant hyperthermia, but the authors proposed a novel mechanism engaging LTD of TRPV1-positive inhibitory neurons. How this postsynaptic TRPV1 is tonically stimulated under pathologic conditions remains to be elucidated.

## 5. Unsolved Issues

Because the studies on the contribution of TRPV1 to the transmission of spinal pain circuit are relatively in their early stage, much of biological information is still unavailable. Here, we focus on several important issues as follows.

### 5.1. Changes in Spinal TRPV1 Expression under Pathologic Conditions

Detailing how dynamically the spinal TRPV1 expression alters depending on the pathologic process may provide more sophisticated explanation of spinal pain mechanisms and help fine-tuning analgesic strategies. As mentioned above, a promising result from TRPV1 expression in the spinal cord has been demonstrated by Kanai et al. [16]. In their CCI rat model, TRPV1 expression of the ipsilateral superficial dorsal horns was gradually elevated for two weeks. This expression location indicates an increase in TRPV1 in the presynaptic terminals by neuropathic insult, supporting the enhanced capsaicin sensitivity of the central terminals in the novel trigeminal CCI neuropathy model [35]. Preceding Kanai et al.'s observation, there were several other similar observations in inflammatory pain model. Tohda et al. detected elevated TRPV1 levels in the dorsal horn presynaptic regions in a carrageenan-inflammation model [53]. In a relatively chronic inflammation model using complete Freund's adjuvant, despite a statistically ambiguous elevation on days from 1 to 2 after injection, over 50% increased TRPV1 levels were maintained for 1 to 3 weeks [6, 8]. After 3 weeks, the expression normalized. The increase may depend on the mediator effect including NGF, which is similar to well-known mechanisms for increases in TRPV1 expression of the peripheral terminals [9]. Therefore, the heightened contribution of presynaptic TRPV1 to pain amplification is predictable. However, information on TRPV1 dynamics of interneurons or projection neurons in the presence or absence of injury is still lacking.

### 5.2. Internal Signaling Cascades

Although TRPV1 is known as a major heat sensor in the body, its thermosensory functions and related thermoregulatory feedback mechanisms are unlikely to be exerted in the spinal cord because this region only experiences a limited range of temperatures, close to the core body temperature. Evidence mentioned above suggests that pharmacological antagonism for TRPV1 in the spinal cord does not cause thermal effects. However, that situation leads to another question: What conditions else activate TRPV1 there? Inflammatory peptides including TNF-*α*, which was tested by Xu et al. [34], and neurotransmitters such as 5-HT, which proved to be a descending modulator, may utilize TRPV1 for a downstream effector. For the TNF-*α*-TRPV1 signaling axis, hypotheses of prostaglandin production for increased TRPV1 activity and extracellular signal-regulated kinase (ERK) activation for elevated TRPV1 expression were raised by studies on DRG neurons [38, 54]. Extracellular prostaglandins are known to enhance TRPV1 sensitivity via phosphorylation by protein kinase A or C through their G-protein-coupled receptor-mediated signaling [55], but it remains elusive whether this paracrine signaling cascade also works for TNF-*α* axis. Even if this prostaglandin-mediated mechanism is true, the mechanism can only sensitize but not activate TRPV1. By this sensitization, heat threshold for TRPV1 activation might descend around the core body temperature. In addition, while only ERK itself has been shown to participate, there has not been sufficient clarification of the further downstream signal in this pathway. Moreover, it also needs to be explored in the presynapse whether the releases of neurotransmitter vesicles are facilitated simply by increased intracellular Ca^2+^ ions through TRPV1 opening-induced depolarization, or other unknown molecular downstream signals are involved.

### 5.3. Interactions with Other Ion Channel Components

The 5-HT3 receptor is a major receptor that receives descending excitatory input from RVM [35]. Interestingly, 5-HT3 receptor is a cation channel that may be functionally redundant regarding the TRPV1 outcome to depolarize the presynaptic area. Whether these two cation channels are additive or otherwise functionally or physically coupled for a synergistic action, as shown in recently published observations at the peripheral terminals about TRPV1-TRPA1 and TRPV1-anoctamin cooperation [56, 57], requires further examination.

Transient receptor potential ankyrin subtype 1 (TRPA1) is comparable to TRPV1 in terms of the importance of covering pain modalities and transductory roles to initiate nociceptor depolarization in the periphery [58]. If both TRP channels share central locations of their expressions, their redundancy, cooperativity, or compensation in the roles in synaptic transmission could not be ignorable. Indeed, the presynaptic facilitative role of TRPA1 in the spinal cord has been addressed [59]. The effects of antagonistic challenges against spinal TRPA1 function were examined and the treatment displayed analgesic outcomes in diverse pain models including SNL, rapid eye movement sleep deprivation, capsaicin-paw injection, formalin-paw injection, and diabetic neuropathy [60–62]. It was further demonstrated that the pain-facilitating effect of descending excitatory inputs from RVM stimulation or spinal cord 5-HT3 receptor activation was all blunted by TRPA1 antagonism [62]. Unlike TRPV1, the analgesic mechanism does not seem to employ the postsynaptic GABAergic disinhibition mechanism [62], which seems to be inconsistent when comparing the earlier and the most recent observations that spinal TRPA1 activation facilitates not only the frequency and amplitude of sEPSCs but also those of sIPSCs [59, 63].

### 5.4. Contribution of Endogenous TRPV1 Activators?

As mentioned, Kim et al. [26] proposed that TRPV1 activation induces LTD in GABAergic interneurons. However, they only conducted external capsaicin administration for TRPV1 activation. Although this can form analgesic proof of concept from a therapeutic viewpoint, it is still poorly understood why the GABAergic neurons need to express TRPV1 regarding their ordinary transmission and what naturally stimulates TRPV1 in neuropathic conditions. Regarding the polymodality of TRPV1, the heat does not seem to be the only candidate. Kim et al. suggested that 12(S)-hydroperoxyeicosatetraenoic acid (12(S)-HPETE) may be a natural TRPV1 activator candidate. Gibson et al. [64] demonstrated that anterograde metabotropic glutamate receptor activation results in postsynaptic 12(S)-HPETE production and that this lipoxygenase metabolite retrogradely diffuses and activates presynaptic TRPV1, causing LTD in the hippocampal CA1 synapses. Application of this paradigm to the spinal cord may narrow down the candidate mechanisms to a GPCR-lipoxygenase cascade but measurement of which substances in the spinal cord are a major metabolite is still required, for example, hepoxilins and hydroxyoctadecadienoic acids [25, 42, 65, 66]. TRPA1 also has a wide spectrum for sensing endogenous reactive substances the levels of which are frequently elevated under injury conditions in tissues or within active synapses. Those are reactive oxygen and nitrogen species and lipid peroxidation products that are known to covalently bind and activate TRPA1 [58, 67]. Generation of these pathologic substances in the spinal cord may facilitate TRPA1-mediated transmission. Normalizing imbalanced local production of atypical excitatory substances for TRP channels by developing specific enzyme inhibitors might be another analgesic strategy to utilize the central TRP channel-mediated synaptic mechanism.

## 6. Extended Questions

Stemming from the recent accomplishments in spinal TRPV1 research, unexplored mechanisms connecting newly uncovered TRPV1's roles and related hypotheses are being raised.

### 6.1. Antagonism by Local Gene Editing

Like pharmacological approaches, gene editing strategies often promote understanding of a mechanism and also offer therapeutic insight. A series of studies using local RNA interference techniques have given new lines of firm evidence for the roles of TRPV1 in pathologic pain progress and also for its action in the spinal cord [68–70]. As tools to improve the efficiency of interfering gene delivery and to lessen safety concerns are being developed, expectancy about future utility of gene editing therapies for chronic pain modulation is currently forming [71, 72]. In the last year, Hirai et al. showed that intrathecal administration of adenoassociated virus serotype 9 (AAV9) vector carrying short-hairpin RNA (shRNA) against TRPV1 resulted in long-term suppression of thermal hyperalgesia in a mouse spared nerve injury model [73]. Viral delivery may confer long-term stable generation of shRNA and limited exposure to immune protection mechanisms outside of the CNS region may help time scale of the effect further since shRNAs in themselves have tough permeability to blood brain barrier (BBB). The confinedness by intrathecal injection may also reduce potential adverse effects from nontarget tissues. Interestingly, despite spinal targeting, only thermal hyperalgesia, not mechanical or cold allodynia, was blunted, which typically occurs in DRG-specific TRPV1 impairment. Conversely, the parameters of shRNA abundance and TRPV1 mRNA reduction were significantly better in the spinal cord than in the DRGs. Differential translational compensation or the presence of TRPV1 isotypes free from the target sequence may be conceivable regarding the broad analgesic spectrum of the above circuit research.

### 6.2. Glial Involvement

Since TRPV1 expression is absent in glial components, it is not likely that the effects of pharmacological modulations of spinal TRPV1 have a direct link to glial contributions. However, in a follow-up study of Kim et al. [35], the Wei lab demonstrated indirect participation of TRPV1 [74]. Although they did not measure TRPV1 activity, Guo et al. repeated presynaptic stimulation by mimicking activation of the descending 5-HT pathway, which subsequently activated microglia and astrocytes. By showing pharmacological and histological evidence, they suggested that fractalkine released from the presynapses stimulates microglia and then interleukin-18 from activated microglia boosts astrocyte function, from which released interleukin-1*β* finally enhances excitability of the dorsal horn neurons by inducing phosphorylation of an N-methyl-D-aspartate receptor subunit. It is currently unexplored whether the presynaptic TRPV1 activation is linked with this process.

### 6.3. NO Other Plastic Players?

Nitric oxide (NO) is of general importance as a retrograde signal for modifying brain synaptic strength. In pain synapses, postsynaptic NO plays a central role for presynaptic activation of the guanylyl cyclase-cyclic guanosine monophosphate- (cGMP-) protein kinase G pathway [75, 76]. Interestingly, TRPV1 activity seems to be tolerant of PKG action or even downregulated by PKG [77, 78]. Not only direct PKG action, but also effects of known substrates of PKG, for example, inositol trisphosphate receptor and myosin light-chain kinase, appear to be independent of the amplification of TRPV1 activity. Calcium/calmodulin-dependent protein kinase II (CAMKII) plays a crucial role in AMPA receptor facilitation in the postsynapse. It has been reported that phosphorylation of TRPV1 of DRG neurons by CAMKII is important for maintaining its sensitivity to ligands, which can be explored regarding the postsynaptic paradigm [79]. *μ*-opioid receptor-induced LTP in the spinal cord appears to occur in TRPV1-positive presynapses and it might be a future issue whether changes in TRPV1 activity in the central terminal are practically involved in opioid signaling [80].

### 6.4. Central Adverse Effects?

Two of many reasons why peripheral TRPV1 has received much attention from industry in the decade since its gene discovery seem to be its polymodality integrating painful inputs with diverse qualities and its peripheral location. For the latter, a central advantage of targeting peripheral TRPV1 is that its ligand avoids the adverse effects of the CNS when it is designed to be BBB-impenetrable. However, assuming that the aim is CNS administration and considering the novel nociceptive roles of TRPV1 in the spinal circuit, one may need to conceive similar adverse situation as already observed or predicted for other CNS analgesic candidates because the target is possibly expressed in other central regions and may have differential actions.

As mentioned above, the bulbospinal circuit receives descending input from periaqueductal gray (PAG) and confers descending excitatory inputs as well as inhibitory ones [35, 81]. Earlier, McGaraughty et al. demonstrated that TRPV1 activation of dorsal PAG gave a hyperalgesic phase via the RVM circuit [82]. However, in ventrolateral PAG (VL-PAG), the neighboring region, it has been suggested that when *μ*-opioid receptor is simultaneously activated TRPV1 activation contributes to facilitation of glutamatergic interneuronal activity, which offers GABAergic inhibition of descending excitatory ON cells in the RVM circuit, leading to a reduction of nociceptive transmission in the spinal cord [83]. Decreased evoked IPSCs, increased miniature IPSCs and EPSCs, and contribution of the cannabinoid receptor have also been proposed by a different intra-PAG recording study [84]. Because this circuit was examined only under acute pain conditions, the analgesic aspect of TRPV1 action in VL-PAG needs to be further addressed for pathologic pain conditions.

Exploring the mechanism of analgesic effects of acetaminophen, researchers have found out that TRPV1 activation in the central nervous system is involved [85, 86]. When acetaminophen is systemically administered, its metabolites are formed in the brain by the action of fatty acid amide hydrolases. Those appear to directly activate brain TRPV1, leading to analgesia in formalin-induced pain and acute thermal or mechanical pain. Antagonism by intracerebroventricular injection of an antagonist and virtual localization of the drug using methylene blue injection argue that supraspinal TRPV1, but not spinal cord TRPV1, may participate in the central analgesic action. Interestingly, some of electrophilic and toxic metabolites produced during the acetaminophen metabolism, different from the metabolites that activate TRPV1, were reported to activate spinal presynaptic TRPA1, resulting in acute antinociception via subsequent inactivation of adjacent presynaptic voltage-gated Na^+^ and Ca^2+^ channels [87].

Other adverse situations due to the potential presence of TRPV1 in CNS regions might be possible. For example, whether inadvertent diffusion of an antagonist in the ventricular regions may enable access to untargeted areas and affect other brain functions including memory or mood needs to be carefully evaluated [64, 88, 89].

### 6.5. Agonistic Challenge as a Therapeutic Strategy?

Whether TRPV1 activation can gain a therapeutic advantage is conceivable. Specific delivery of local anesthetics to TRPV1-positive nociceptor is being considered as a novel analgesic strategy [90, 91]. Some membrane-impermeable hydrophilic derivatives of lidocaine species are able to permeate into neurons through dilated TRPV1 pore when TRPV1 is activated and once inside, they can block voltage-gated Na^+^ channels, with or without their permeant blockade of TRPV1 itself. TRPV1-negative neurons should be inert to the blocking effects of these drugs since the drugs can be admitted only through TRPV1, which may allow avoidance of common adverse effects of the local anesthetics including numbness and motor defects via interfering nonpain pathways. Such approaches of specific application may be taken into consideration in the future for modulation of spinal cord TRPV1.

Among TRPV1-targeting pain therapies, only topical capsaicin treatment is currently available in clinics. The rationale is based on the functional incapacitation of the peripheral sensory terminals by agonist-induced TRPV1 desensitization and mitochondrial permeability transition which leads to terminal ablation [79, 92]. The same mechanism might be possible in the spinal region: analgesia via defunctionalization of TRPV1-specific pre- and postsynapses by agonist-induced effects. However, as mentioned, intrathecal administration of TRPV1 agonists resulted in some pain phenotypes in animal studies [25, 26]. This appears to be predictable because it is an unavoidable side effect that topical capsaicin application in its early treatment stage evokes pain via initial TRPV1 activation in humans. One possible option to overcome this hurdle is currently thought to be a substitution by a nonpungent capsaicin analogue free from the initial pain induction [93]. Although such a class of TRPV1 agonists were recently developed, they have not been thoroughly tested regarding their analgesic effects.

## 7. Conclusion

In the early stage, more attention was given to the sensory involvement of TRPV1 in the peripheral terminal of the nociceptor neurons to harmful environments and to its contribution to neurogenic inflammation [94, 95]. However, revisiting traditional capsaicin pharmacology, assessments regarding TRPV1 expression patterns and their dynamics in diverse neural regions have provided clues of other nociceptive roles for TRPV1. Particularly, recent accumulation of knowledge on TRPV1 functions in the spinal presynaptic and postsynaptic locations connecting to exacerbation mechanisms for neuropathic pain has begun to address its roles in the central nervous system. This heightened understanding and new hypotheses also appear to raise the possibility of developing new proof of concept targeting spinal TRPV1. Such attempts for new approaches could be extended to other CNS locations and other polymodal TRP channels such as TRPA1. Further analyses regarding the role of TRPV1 in plastic changes for pain synapses at the individual level including supraspinal circuits will shed light on the collective contribution to exacerbation of pathologic pain and its analgesic utility.
